# Association of the FDA Amendment Act with trial registration, publication, and outcome reporting

**DOI:** 10.1186/s13063-017-2068-3

**Published:** 2017-07-18

**Authors:** Adam T. Phillips, Nihar R. Desai, Harlan M. Krumholz, Constance X. Zou, Jennifer E. Miller, Joseph S. Ross

**Affiliations:** 1 Division of Cardiovascular Medicine, Department of Internal Medicine, Beth Israel Deaconess Medical Center, Boston, MA USA; 2 Section of Cardiovascular Medicine, Department of Internal Medicine, Yale School of Medicine, New Haven, CT USA; 3 Center for Outcomes Research and Evaluation, Yale-New Haven Hospital, New Haven, CT USA; 4 Robert Wood Johnson Foundation Clinical Scholars Program, Department of Internal Medicine, Yale School of Medicine, New Haven, CT USA; 5 Department of Health Policy and Management, Yale School of Public Health, New Haven, CT USA; 6 Yale School of Medicine, New Haven, CT USA; 7 Division of Medical Ethics, Department of Population Health, NYU School of Medicine, Bioethics International, New York, NY USA; 8 Section of General Medicine, Department of Internal Medicine, Yale School of Medicine, P.O. Box 208093, New Haven, CT 06520-8093 USA

**Keywords:** Clinical trials, Publications, Drug approval, United States Food and Drug Administration

## Abstract

**Background:**

Selective clinical trial publication and outcome reporting has the potential to bias the medical literature. The 2007 Food and Drug Administration (FDA) Amendment Act (FDAAA) mandated clinical trial registration and outcome reporting on ClinicalTrials.gov, a publicly accessible trial registry.

**Methods:**

Using publicly available data from ClinicalTrials.gov, FDA documents, and PubMed, we determined registration, publication, and reporting of findings for all efficacy trials supporting FDA approval of new drugs for cardiovascular disease and diabetes between 2005 and 2014, before and after the FDAAA. For published trials, we compared the published interpretation of the findings (positive, equivocal, or negative) with the FDA reviewer’s interpretation.

**Results:**

Between 2005 and 2014, the FDA approved 30 drugs for 32 indications of cardiovascular disease (*n* = 17) and diabetes (*n* = 15) on the basis of 183 trials (median per indication 5.7 (IQR, 3–8)). Compared with pre FDAAA, post-FDAAA studies were more likely to be registered (78 of 78 (100%) vs 73 of 105 (70%); *p* < 0.001), to be published (76 of 78 (97%) vs 93 of 105 (89%); *p* = 0.03), and to present findings concordant with the FDA reviewer’s interpretation (74 of 76 (97%) vs 78 of 93 (84%); *p* = 0.004). Pre FDAAA, the FDA reviewer interpreted 80 (76%) trials as positive and 91 (98%) were published as positive. Post FDAAA, the FDA reviewer interpreted 71 (91%) trials as positive and 71 (93%) were published as positive.

**Conclusions:**

FDAAA was associated with increased registration, publication, and FDA-concordant outcome reporting for trials supporting FDA approval of new drugs for cardiovascular disease and diabetes.

**Electronic supplementary material:**

The online version of this article (doi:10.1186/s13063-017-2068-3) contains supplementary material, which is available to authorized users.

## Background

The US Food and Drug Administration (FDA) approves new drugs based on clinical evidence, requiring “adequate and well controlled investigations”, to demonstrate safety and efficacy [1]. The FDA suggests that drug sponsors provide two or more “pivotal” efficacy trials [2]— typically large, randomized, controlled trials—as well as “non-pivotal” trials that provide additional insights into drug efficacy and safety. These trials provide the earliest evidence to inform use of newly approved drugs, and as such it is critical that their findings are disseminated through the peer-reviewed medical literature to inform clinical care.

Among recently approved drugs, studies have shown that nearly 90% of pivotal clinical trials were published [3, 4]. However, older studies raised concerns about the completeness, and accuracy, of the published evidence [5]. For instance, one study examining clinical trials supporting FDA approval of antidepressants between 1987 and 2004 demonstrated that only 70% of those submitted to the FDA had been published, and that trials with positive findings were more likely to be published [5]. Furthermore, some trials interpreted by the FDA to have negative or neutral findings were published to convey positive findings. These practices, known as selective publication and selective outcome reporting, distort the published evidence. However, in 2007, the US FDA Amendment Act (FDAAA) was enacted, mandating clinical trial registration and results reporting on ClinicalTrials.gov, a publicly accessible clinical trial registry established by the National Institutes of Health, for all ongoing and forthcoming trials of FDA-regulated products [6]. The extent to which the FDAAA mitigated selective registration, publication, and outcome reporting is not known.

Accordingly, our objective was to determine registration, publication, and reporting of findings for all efficacy trials supporting FDA approval of new drugs for cardiovascular disease and diabetes between 2005 and 2014, before and after the FDAAA. We focused our study on drugs in cardiovascular disease and diabetes because they are among the most well studied and widely prescribed drugs in the US market and represent a large proportion of all newly approved drugs [7]. In addition, for published trials, we compared the published interpretation of the trial findings with the FDA reviewer’s interpretation.

## Methods

### Data sources

We obtained information on FDA-approved drugs using the Drugs@FDA database, a publicly available data source that includes regulatory actions and labeling changes for all currently approved drugs [8]. For each drug, there are hyperlinks to medical and statistical reviews prepared by the FDA that include all clinical data relevant to the new drug application. All Drugs@FDA reviews were downloaded as of August 2016.

### Drug sample

**Fig. 1:**
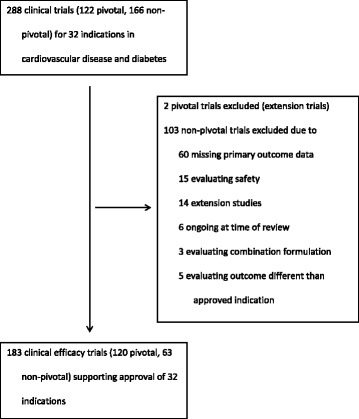
Sample construction of novel therapeutic agents in cardiovascular disease and diabetes approved by the US Food and Drug Administration between 2005 and 2014

We identified a sample of novel therapeutics (i.e., new molecular entities or novel biologic drugs) first approved by the FDA between January 1, 2005, and December 31, 2014, excluding generic drugs, reformulations, and combination therapies of non-novel therapeutic agents (Fig. 1). We limited our sample to drugs in the therapeutic area of cardiovascular disease and diabetes mellitus as defined by the World Health Organization’s Anatomic Therapeutic Classification system (http://www.whocc.no/atc_ddd_index/), contextualized for clinical relevance, as reported previously [7]. All drugs were characterized as having received either priority or standard review by the FDA and by whether or not they were designated with orphan status.

### Selection of clinical trials

The Drugs@FDA database contains documents for all regulatory actions related to new drug approvals, including medical reviews with clinical data relevant to the drug of interest, summarized by the FDA reviewer. The medical reviews include an overview of safety and efficacy, an outline of the sources of clinical data, integrated summaries of safety and efficacy, and, where relevant, review of individual clinical trials.

Within each document is a section titled “Sources of Clinical Data” which includes a “Table of Studies/Clinical Trials” subsection. From this table we identified all phase 2 and phase 3 clinical trials examining drug efficacy. We excluded phase 1 trials and trials missing primary outcome definitions or results, conducted to exclusively evaluate safety, ongoing at the time of approval, extensions of trials described previously in the review, and expanded-access trials.

### Characterization of clinical trials

Trials supporting approvals were categorized as pre FDAAA if the primary completion date was before December 26, 2007 (date the policy took effect), as indicated on ClinicalTrials.gov, FDA documents, or peer-reviewed publications; all other trials were categorized as post FDAAA.

In addition, we categorized trials by whether they were designated as pivotal or nonpivotal, phase 2 or phase 3, and by several design characteristics: randomization (yes/no), blinding and allocation concealment (double blinded yes/no), comparator type (active yes/no), trial size (more than 500 patients yes/no), trial duration (longer than 12 weeks yes/no), and trial quality (Jadad score ≥ 3 yes/no) [9] We also extracted primary outcome definitions and results as well as the overall trial findings of the FDA reviewer (positive, equivocal, negative). Trials were classified as equivocal if the FDA reviewer judged the trial to lack significant findings on the primary outcome but have significant findings on several secondary outcomes, consistent with prior literature [5]. For dose-ranging trials (trials for which patients were assigned to one of several doses or placebo), we used the overall decision of the FDA reviewer.

### Identification of trials registered on ClinicalTrials.gov and published in the peer-reviewed literature

For each trial described in FDA documents, we conducted a comprehensive search of ClinicalTrials.gov and PubMed in August 2016 to identify any corresponding registered trial or publication, respectively. Our search strategy used a combination of drug name, sponsor company, dosage groups, sample size, active comparator (if used), and duration, an approach used in prior research [3, 10, 11].

### Comparison of published trials and FDA-submitted trials

Data were abstracted from the FDA summaries and PubMed publications by one author (ATP) and validated by a second author (CXZ). Any differences in findings were reconciled by consensus. For each trial described in FDA documents for which a publication in the peer-reviewed literature was identified, we compared the reported primary outcome definition and results, and the overall study interpretation, between the two sources. The overall interpretation was categorized as positive, equivocal, or negative based on the FDA officer’s language in the medical review and the author’s language in the conclusion of the publication; the FDA and publication interpretation were then categorized as concordant or discordant. For example, the Dronedarone ADONIS study was characterized as equivocal in the FDA documents and positive in the peer-reviewed publication because the FDA reviewer wrote “Dronedarone … significantly delayed the first AF/AFL recurrence. It also delayed the symptomatic first recurrence. However, these reviewers disagree with the Sponsor that dronedarone significantly reduced ventricular rate at the time of first recurrence” [12], whereas the authors in the peer-reviewed publication concluded that “Dronedarone was significantly more effective than placebo in maintaining sinus rhythm and in reducing the ventricular rate during recurrence of arrhythmia” [13].

### Statistical analysis

We determined the overall rate of ClinicalTrials.gov registration and publication, and then stratified studies by type, drug, and design characteristics, using chi-square and Fisher exact tests as appropriate to compare rates of registration and publication across groups. We then categorized trials by concordance between the FDA review and publication to compare rates of concordance among trials stratified by type, drug, and design characteristics, again using chi-square and Fisher exact tests as appropriate. Finally, we categorized trials by the FDA reviewer’s interpretation and determined the publication rate for studies deemed positive, negative, or equivocal. For each categorization, we stratified trials as pre or post FDAAA. Analyses were performed using Microsoft Excel 15.11.2 (Microsoft Corporation, Redmond, WA, USA) and EpiInfo 3.0 (Centers for Disease Control and Prevention, Atlanta, GA, USA). The PRISMA checklist is presented in Additional file 1.

## Results

Between 2005 and 2014, the FDA approved 30 novel therapeutics for 32 indications for the treatment of cardiovascular disease (17 (53%)) and diabetes (15 (47%); Table 1). Among these, three (9%) indication approvals were designated orphan status and six (19%) were designated priority review status.

**Table 1:** Novel therapeutic agent indications approved by the US FDA for cardiovascular disease and diabetes between 2005 and 2014

Novel therapeutic agent indication approvals (*n* = 32)	Number (%)
Orphan status^a^	
Yes	3 (9)
No	29 (91)
FDA review pathway^a^	
Priority	6 (19)
Standard	26 (81)
Therapeutic area^b^	
Cardiovascular disease	17 (53)
Diabetes mellitus	15 (47)

*FDA* Food and Drug Administration

^a^FDA designation

^b^As defined by the World Health Organization’s Anatomic Therapeutic Classification system (http://www.whocc.no/atc_ddd_index/), contextualized for clinical relevance

We identified a total of 288 clinical trials (122 pivotal, 166 nonpivotal) supporting these 32 indication approvals, of which 183 (120 pivotal, 63 nonpivotal) met our inclusion criteria (Fig. 1), a median of 5.7 (IQR, 3–8) trials per approval. Of the 183 clinical trials identified, 78 (43%) were post FDAAA, 151 (83%) were phase 3, 176 (96%) were randomized, 160 (87%) were double blinded, 75 (41%) had an active comparator, 116 (63%) were longer than 12 weeks in duration, 93 (51%) had more than 500 patients, and 175 (96%) had Jadad score ≥ 3.

### Trial registration and publication

Among the 183 trials, 151 (83%) were registered on ClinicalTrials.gov, 99 (54%) had posted results, and 169 (92%) were published in the peer-reviewed literature. Trials registered on ClinicalTrials.gov (145 of 151 (96%)) were more likely to be published than those not registered (24 of 32 (75%); *p* = 0.0005) (Table 2). Pre FDAAA, certain trial characteristics were associated with likelihood of registration and publication, including double-blind design, enrollment > 500 patients, and Jadad score ≥ 3 (Table 3). Post FDAAA, all trials were registered, and trial duration > 12 weeks was associated with likelihood of publication (Table 4). Additionally, post-FDAAA trials were more likely to be registered (78 of 78 (100%) vs 73 of 105 (70%); *p* < 0.001) and to post results (70 of 78 (90%) vs 30 of 106 (28%); *p* = 0.0001) on ClinicalTrials.gov. They were also more likely to be published (76 of 78 (97%) vs 93 of 105 (89%); *p* = 0.03).

**Table 2:** Registration, publication, and interpretation concordance of clinical trials supporting FDA new drug approvals in cardiovascular disease and diabetes between 2005 and 2014, pre and post FDAAA

	Number of studies (%)	Registered, *n* (%)	Odds ratio (95% CI), *p* value	Published, *n* (%)^a^	Odds ratio (95% CI), *p* value	Concordant interpretation, *n*. (%)^a^	Odds ratio (95% CI), *p* value
Overall	183 (100)	151 (83)		169 (92)		152 (90)	
Trial completion date							
Pre FDAAA	105 (57)	73 (70)	–, <0.0001	93 (89)	0.20 (0.04 to 0.94), 0.03	78 (84)	0.14 (0.03 to 0.64), 0.004
Post FDAAA	78 (43)	78 (100)		76 (97)		74 (97)	

*CI* confidence interval, *FDA* US Food and Drug Administration, *FDAA* FDA Amendment Act, – too few events to calculate odds ratio

^a^“Published” includes publication in the peer-reviewed literature. The “Concordant interpretation” column uses “Published” as the denominator to determine percentages

**Table 3:** Registration, publication, and interpretation concordance of clinical trials supporting FDA new drug approvals in cardiovascular disease and diabetes between 2005 and 2014, stratified by study and drug characteristics, pre FDAAA

	Number of studies (%)	Registered*, n* (%)	Odds ratio (95% CI), *p* value	Published, *n* (%)^a^	Odds ratio (95% CI), *p* value	Concordant interpretation, *n* (%)^a^	Odds ratio (95% CI), *p* value
Overall	183 (100)	151 (83)		169 (92)		152 (90)	
Pre FDAAA							
Therapeutic category^b^							
Cardiovascular disease	52 (50)	39 (61)	1.67 (0.72 to 3.89), 0.29	47 (90)	1.43 (0.42 to 4.83), 0.76	40 (85)	1.20 (0.40 to 3.64), 0.78
Diabetes	53 (50)	34 (64)		46 (87)		38 (83)	
FDA review pathway^c^							
Priority review	9 (9)	8 (89)	3.82 (0.46 to 31.85), 0.27	9 (100)	–, 1.00	6 (67)	0.47 (0.11 to 2.08), 0.38
Standard review	96 (91)	65 (71)		89 (93)		72 (81)	
Orphan status^c^							
Yes	2 (2)	2 (100)	–, 1.00	2 (100)	–, 1.00	2 (100)	–, 1.00
No	103 (98)	71 (69)		91 (88)		76 (84)	
Trial designation^d^							
Pivotal	63 (60)	50 (79)	3.17 (1.34 to 7.52), 0.01	57 (90)	1.58 (0.47 to 5.29), 0.54	51 (89)	2.83 (0.91 to 8.80), 0.08
Nonpivotal	42 (40)	23 (55)		36 (86)		27 (75)	
Trial phase							
Phase 2	22 (21)	12 (57)	0.43 (0.16 to 1.14), 0.11	17 (77)	0.31 (0.09 to 1.11), 0.12	17 (100)	–, 0.06
Phase 3	83 (79)	61 (73)		76 (92)		61 (80)	
Randomized design							
Yes	102 (97)	71 (70)	1.15 (0.01 to 13.1), 0.67	92 (90)	18.4 (1.52 to 221.40), 0.04	77 (84)	–, 1.00
No	3 (3)	2 (67)		1 (33)		1 (100)	
Double blinded							
Yes	87 (83)	66 (76)	4.94 (1.70 to 14.36), 0.003	82 (94)	10.4 (2.82 to 36.64), 0.0007	73 (89)	9.73 (2.46 to 38.46), 0.002
No	18 (17)	7 (39)		11 (61)		5 (45)	
Active comparator							
Yes	43 (41)	31 (72)	1.23 (0.52 to 2.89), 0.67	37 (86)	0.66 (0.20 to 2.21), 0.54	28 (76)	0.37 (0.12 to 1.16), 0.09
No	62 (59)	42 (68)		56 (90)		50 (89)	
Trial duration							
≤12 weeks	52 (50)	37 (71)	1.16 (0.51 to 2.67), 0.88	46 (88)	0.98 (0.29 to 3.26), 0.61	41 (89)	2.21 (0.69 to 7.09), 0.26
>12 weeks	53 (50)	36 (68)		47 (89)		37 (80)	
Number of patients in trial							
<500	59 (56)	36 (61)	0.28 (0.15 to 0.93), 0.03	48 (81)	0.19 (0.04 to 0.92), 0.01	41 (85)	1.26 (0.42 to 3.83), 0.78
≥500	46 (44)	37 (78)		45 (98)		37 (82)	
Jadad score							
<3	3 (3)	2 (67)	0.87 (0.07 to 9.99), 1.00	1 (33)	0.05 (0.01 to 0.65), 0.03	1 (100)	–, 1.00
≥3	102 (97)	71 (70)		92 (90)		77 (84)	

*CI* confidence interval, *FDA* US Food and Drug Administration, *FDAA* FDA Amendment Act, – too few events to calculate odds ratio

^a^“Published” includes publication in the peer-reviewed literature. The “Concordant interpretation” column uses “Published” as the denominator to determine percentages

^b^As defined by the World Health Organization’s Anatomic Therapeutic Classification system (http://www.whocc.no/atc_ddd_index/), contextualized for clinical relevance

^c^FDA designation

^d^A trial was considered pivotal if it was the only trial included in the summary or if it was explicitly designated as pivotal; all other trials were nonpivotal

**Table 4:** Registration, publication, and interpretation concordance of clinical trials supporting FDA new drug approvals in cardiovascular disease and diabetes between 2005 and 2014, stratified by study and drug characteristics, post FDAAA

	Number of studies (%)	Registered, *n* (%)	Odds ratio (95% CI), *p* value	Published, *n* (%)^a^	Odds ratio (95% CI), *p* value	Concordant interpretation, *n* (%)^a^	Odds ratio (95% CI), *p* value
Overall	183 (100)	151 (83)		169 (92)		152 (90)	
Post FDAAA							
Therapeutic category^b^							
Cardiovascular disease	21 (27)	21 (100)	–, 1.00	19 (90)	–, 0.07	19 (100)	–, 1.00
Diabetes	57 (73)	57 (100)		57 (100)		55 (96)	
FDA review pathway^c^							
Priority review	9 (12)	9 (100)	–, 1.00	9 (100)	–, 1.00	9 (100)	–, 1.00
Standard review	69 (88)	69 (100)		67 (97)		65 (97)	
Orphan status^c^							
Yes	6 (8)	6 (100)	–, 1.00	6 (100)	–, 1.00	6 (100)	–, 1.00
No	72 (92)	72 (100)		70 (97)		68 (97)	
Trial designation^d^							
Pivotal	57 (73)	57 (100)	–, 1.00	55 (96)	–, 1.00	53 (96)	–, 1.00
Nonpivotal	21 (27)	21 (100)		21 (100)		21 (100)	
Trial phase							
Phase 2	10 (13)	10 (100)	–, 1.00	10 (100)	–, 1.00	10 (100)	–, 1.00
Phase 3	68 (87)	68 (100)		66 (97)		64 (97)	
Randomized design							
Yes	74 (95)	74 (100)	–, 1.00	72 (97)	–, 1.00	70 (97)	–, 1.00
No	4 (5)	4 (100)		4 (100)		4 (100)	
Double blinded							
Yes	72 (92)	72 (100)	–, 1.00	70 (97)	–, 1.00	68 (94)	–, 1.00
No	6 (8)	6 (100)		6 (100)		6 (100)	
Active comparator							
Yes	32 (41)	32 (100)	–, 1.00	32 (100)	–, 1.00	30 (94)	–, 1.00
No	46 (59)	46 (100)		44 (96)		44 (100)	
Trial duration							
≤ 12 weeks	15 (19)	15 (100)	–, 1.00	13 (87)	–, 0.04	13 (100)	–, 1.00
> 12 weeks	63 (81)	63 (100)		63 (100)		61 (97)	
Number of patients in trial							
< 500	31 (40)	31 (100)	–, 1.00	30 (97)	0.65 (0.04 to 10.83), 0.64	30 (100)	–, 0.52
≥ 500	47 (60)	47 (100)		46 (98)		44 (96)	
Jadad score							
< 3	6	6	–, 1.00	6	–, 1.00	6	–, 1.00
≥ 3	72	72		70		68	

*CI* confidence interval, *FDA* US Food and Drug Administration, *FDAA* FDA Amendment Act, – too few events to calculate odds ratio

^a^“Published” includes publication in the peer-reviewed literature. The “Concordant Interpretation” column uses “Published” as the denominator to determine percentages

^b^As defined by the World Health Organization’s Anatomic Therapeutic Classification system (http://www.whocc.no/atc_ddd_index/), contextualized for clinical relevance

^c^FDA designation

^d^A trial was considered pivotal if it was the only trial included in the summary or if it was explicitly designated as pivotal; all other trials were nonpivotal

### FDA reviewer trial interpretation

**Fig. 2:**
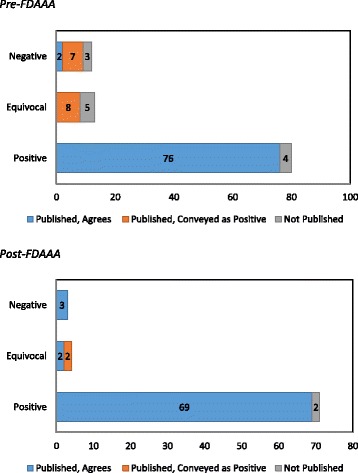
FDA reviewer trial interpretation and publication, along with published interpretation of the trial findings, for novel therapeutic agents in cardiovascular disease and diabetes approved by the US FDA between 2005 and 2014, pre and post the Food and Drug Administration Amendment Act (*FDAAA*). FDA reviewer trial interpretation as positive, equivocal, or negative

**Fig. 3:**
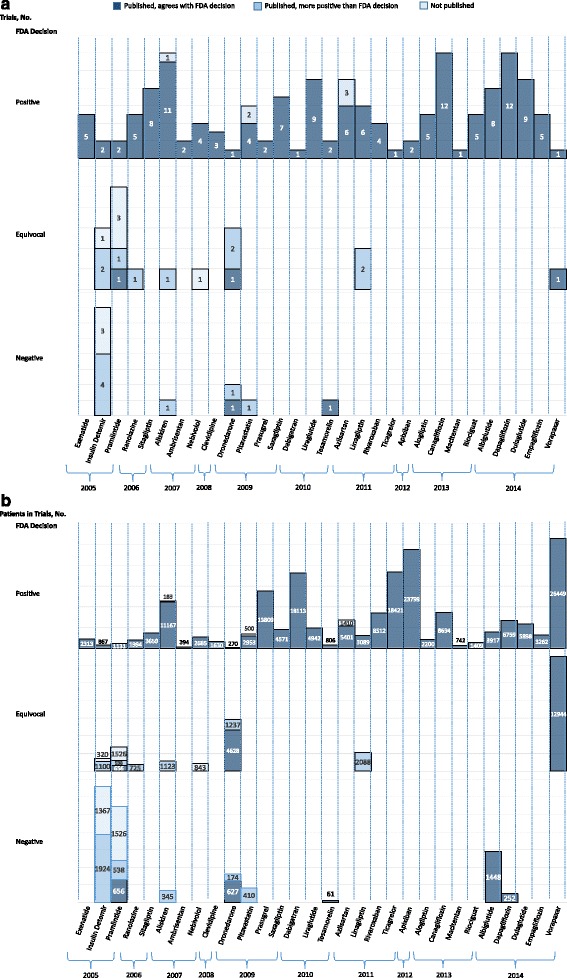
FDA reviewer trial interpretation and publication, along with published interpretation of the trial findings, for all novel therapeutic agents in cardiovascular disease and diabetes approved by the US Food and Drug Administration (*FDA*) between 2005 and 2014, characterized based on the number of trials (**a**) and the number of patients participating in the trials (**b**). FDA reviewer trial interpretation as positive, equivocal, or negative

Among the 105 pre-FDAAA trials, the FDA reviewer characterized 80 (76%) as positive, 13 (12%) as equivocal, and 12 (11%) as negative; whereas among the 78 post-FDAAA trials, the FDA reviewer characterized 71 (91%) as positive, 4 (5%) as equivocal, and 3 (4%) as negative (Fig. 2). Pre FDAAA, trials characterized as positive were more likely to be registered (63 of 80 (79%) vs 10 of 25 (40%); *p* = 0.0005) and published (76 of 80 (95%) vs 17 of 25 (68%); *p* = 0.001) than negative or equivocal trials. The higher publication rate was primarily driven by trials for drugs in diabetes, in which positive trials were more likely to be published (38 of 38 (100%) vs 8 of 15 (53%); *p* = 0.0001) than negative or equivocal trials; trials for drugs in cardiovascular disease had similar rates of publication for positive trials (38 of 42 (90%)) and negative or equivocal trials (9 of 10 (90%); *p* = 0.67). Post FDAAA, all trials were registered and there was no difference in publication rate between positive trials (69 of 71 (97%)) and negative or equivocal trials (7 of 7 (100%); *p* = 0.83; Fig. 3).

### Concordance between publication and FDA reviewer interpretation

Among the 93 pre-FDAAA published trials, 91 (98%) conveyed positive and 2 (2%) negative findings. Of these, 78 (84%) were published in a manner concordant with the FDA reviewer’s interpretation and 15 (16%) were published in a manner that conveyed a more positive interpretation than that of the FDA reviewer. Among the 76 post-FDAAA published trials, 71 (93%) conveyed positive findings, 2 (3%) equivocal findings, and 3 (4%) negative findings. Of these, 74 (97%) were published in a manner concordant with the FDA reviewer’s interpretation and 2 (3%) were published in a manner than conveyed a more positive interpretation than that of the FDA reviewer (see Additional file 2 for details of all discordant interpretations). Overall, no trials were published in a manner that conveyed a more negative interpretation than that of the FDA reviewer.

Rates of concordant interpretation were higher for post-FDAAA trials (74 of 76 (97%)) than pre-FDAAA trials (78 of 93 (84%); *p* = 0.004). Pre FDAAA, rates of concordant interpretation were higher for phase 2 trials and for those with double-blinded design (Table 2). Additionally, positive trials were more likely to have concordant findings (76 of 76 (100%)) than negative or equivocal trials (2 of 17 (12%); *p* = 0.03). Post FDAAA, there were no significant differences in concordance based on drug or trial characteristics. Positive trials, however, were more likely to have concordant findings (69 of 69 (100%)) than negative or equivocal trials (5 of 7 (83%); *p* = 0.007). Overall, trials registered on ClinicalTrials.gov (136 of 145 (94%)) were more likely to have concordant findings than those not registered (15 of 24 (63%); *p* < 0.0001).

## Discussion

The FDAAA, US legislation that mandated clinical trial registration and outcome reporting on ClinicalTrials.gov, was associated with lower rates of selective registration, publication, and outcome reporting among trials supporting FDA approval of new drugs for cardiovascular disease and diabetes between 2005 and 2014. However, there is still room for improvement because 11% of pre-FDAAA trials remain unpublished and there was an inconsistency in outcome reporting between the FDA review and the publication for 16%, as the trials were published in a manner that conveyed a more positive interpretation than the FDA reviewer. Post FDAAA, only 3% of the trials were unpublished, and of those published only 3% were done so with a more positive interpretation than the FDA reviewer. Overall, our study suggests that while nearly one-third of pre-FDAAA trials were not represented completely and accurately in the published literature, only one in 15 post-FDAAA trials were not represented completely and accurately in the published literature.

Three drugs accounted for the vast majority of trials that were unpublished or published with a more positive interpretation than that of the FDA reviewer (all pre FDAAA): dronedarone, insulin detemir, and pramlintide. Dronedarone is approved to reduce the risk of hospitalization in patients with atrial fibrillation. Three trials, two published in the *New England Journal of Medicine* (EURIDIS and ADONIS) and one in the *American Heart Journal* (ERATO), had conflicting interpretations in the FDA review and the journal article. The FDA interpretations of the EURIDIS and ADONIS trials were both considered equivocal because each demonstrated that dronedarone delayed the time to first recurrence of atrial fibrillation, but the ventricular rate during recurrence was unchanged from placebo; in contrast, the interpretations in the publications were considered positive because both concluded that dronedarone reduced the ventricular rate during recurrence. The FDA interpretation of the ERATO trial was considered negative because it demonstrated lack of efficacy for a clinically important secondary endpoint, exercise tolerance, whereas the publication had a positive interpretation based on a reduction in ventricular rate during first recurrence, a surrogate marker of disease.

Insulin detemir is approved to improve glycemic control in patients with diabetes. The FDA interpretation of several trials was considered negative or equivocal because of the variable amount of short-acting insulin used by participants, making it difficult to determine the relative efficacy of insulin detemir vs other long-acting insulin formulations. In contrast, the interpretations in the publications were positive because they demonstrated better balance of glucose control and variability, and less weight gain. Pramlintide, similarly, is approved to improve glycemic control in patients with diabetes. In one trial, the FDA interpretation was negative because the trials failed to show efficacy for the prespecified primary endpoint of reduction in hemoglobin a1c at 52 weeks; in contrast, the journal article was positive because it demonstrated a reduction in hemoglobin a1c at 13 weeks. For busy clinicians reading only the abstract of a journal article without exposure to a deep analysis of the data, these altered interpretations can influence utilization of the medication.

Our findings show post-FDAAA improvement compared to Turner’s landmark paper describing selective publication and outcome reporting among studies supporting FDA approval of antidepressants between 1987 and 2004, which demonstrated that only 70% of clinical trials had been published, but that 94% of published trials conveyed positive findings, despite the fact that only 51% of the trials submitted to the FDA had been positive [5]. Pre FDAAA, we found that three of four trials submitted to the FDA had positive results, 95% of which were published; and among those characterized by the FDA as equivocal or negative, 92% were either unpublished or published in a way that conveyed positive findings. The published literature gives the appearance that 98% of the trials are positive, despite the fact that only 76% of the trials submitted to the FDA were interpreted by the FDA reviewer as positive. Post FDAAA, we found that 91% of trials submitted to the FDA were positive, 97% of which were published; and among those characterized by the FDA as equivocal or negative, 29% were either unpublished or published in a way that conveyed positive findings. The published literature gives the appearance that 93% of the trials are positive, similar to the 91% interpreted as positive by the FDA reviewer.

Registration within ClinicalTrials.gov improved after implementation of the FDAAA, such that nearly all of the trials submitted to support FDA approval that were subject to the 2007 FDAAA regulations were registered on ClinicalTrials.gov, but only half have reported results thus far [14]. These findings are slightly more encouraging than prior research, which demonstrated that only 41.5% of trials subject to the FDAAA reported results within 5 years of study completion [15]. The improvement in reporting of results may be due to increasing familiarity with the process among clinical trialists, increasingly ubiquitous registration requirements for journal publication, and overall culture change regarding clinical trial transparency.

There are several limitations to our study. First, we only looked at trials supporting FDA approval of drugs used for the treatment of cardiovascular disease and diabetes. Our results may not be generalizable to all clinical areas, because prior research has shown that publication rates for pivotal trials supporting cardiovascular disease and diabetes approvals are lower than in other therapeutic areas [3], raising the possibility that there was a greater opportunity for improvement. Our findings should be confirmed for FDA approvals in other therapeutic areas. Second, our analysis was focused on trials providing efficacy data to support FDA approval and is not generalizable to trials focused on safety. Third, we split our observation period into two periods based on the adoption date of FDAAA, but there was likely a more gradual improvement than that implied by our analysis, and other factors, such as improved awareness and knowledge of clinical trial requirements over time among both trialists and journal editors, may have contributed to the improvement in registration, publication, and concordance rates. Fourth, we did not determine whether the results reported on ClinicalTrials.gov were concordant with the results presented in the FDA documents and published literature; this was outside the scope of this review. Fifth, for trials determined to be unregistered or unpublished we did not contact sponsor companies for confirmation. Finally, because our study was observational in nature, we are unable to draw causal conclusions about the impact of the FDAAA on trial registration, publication, and interpretation concordance.

## Conclusions

The 2007 FDAAA was associated with higher rates of clinical trial registration on ClinicalTrials.gov, publication, and concordance between interpretation of findings in the FDA documents and published literature. However, many older trials remain unpublished or published with a more positive conclusion than that of the FDA review, suggesting a clear need to improve the historical integrity of the clinical research enterprise. Further efforts to improve accessibility of older and negative clinical trials, such as online FDA resources, within drug labels, and negative trial journals or repositories, are needed to ensure complete and timely dissemination of clinical research.

## Additional files

Additional file 1: PRISMA checklist. (DOC 63 kb)

Additional file 2: Table S1. Presenting the clinical trials supporting FDA approvals of drugs in cardiovascular disease and diabetes that were published in the biomedical literature in a manner that was discordant with the FDA reviewer’s interpretation. (DOCX 32 kb)

## Abbreviations

FDA: Food and Drug Administration
FDAAA: Food and Drug Administration Amendment Act
