# Supplementary material for: Association of the FDA Amendment Act with trial registration, publication, and outcome reporting
Source: Trials. 2017 Jul 18;18:333. doi: 10.1186/s13063-017-2068-3 (PMC5516301; doi:10.1186/s13063-017-2068-3)
Supplement: Supplementary file 2 — Presenting the clinical trials supporting FDA approvals of drugs in cardiovascular disease and diabetes that were published in the biomedical literature in a manner that was discordant with the FDA reviewer’s interpretation. (DOCX 32 kb) [file 13063_2017_2068_MOESM2_ESM.docx]

Additional file 2: Appendix

**Table S1.** Clinical Trials Supporting FDA Approvals of Drugs in Cardiovascular Disease and Diabetes That Were Published in the Biomedical Literature in a Manner That Was Discordant With the FDA Reviewer’s Interpretation.

| Study ID | Study Type | Primary Efficacy Outcome | FDA Summary- and Publication-Reported Results | FDA Reviewer Interpretation | Publication Interpretation |
| --- | --- | --- | --- | --- | --- |
| Aliskiren 2203 | Pivotal | Change in mean sitting diastolic blood pressure (Aliskiren versus placebo) | Placebo: -8.6 mmHg; Aliskiren 75 mg: -10.3 mmHg (P=0.052); Aliskiren 150 mg -10.3 mmHg (P=0.051); Aliskiren 300 mg -12.3 mmHg (P<0.0001) | **Equivocal**: “Weakly supportive” results, and only at the highest dosage. | **Positive**: “Antihypertensive efficacy and placebo-like tolerability.”[1] |
| Aliskiren 2324 | Non-pivotal | Change in mean 24 hour systolic blood pressure (Aliskiren versus Lisinopril) | Aliskiren 75 mg: -8.4 mmHg; Aliskiren 150 mg -7.1 mmHg; Aliskiren 300 mg -8.7 mmHg; Lisinoprol 10 mg -10.2 mmHg | **Negative**:  Fails to show dose response, slightly less effective than lisinopril 10 mg. | **Positive**:  Effective 24 hour blood pressure lowering (compared endpoint reductions to baseline instead of to lisinopril).[2] |
| Dronedarone ADONIS | Pivotal | Time between randomization and first atrial fibrillation/flutter recurrence (Dronedarone versus placebo) | Drondarone 800 mg: 158 days; placebo: 59 days; log-rank test P valve 0.0017 | **Equivocal**: Delayed time to recurrence, but no clinically significant reduction in ventricular rate at first recurrence | **Positive**:  Delayed time to recurrence, and reduced ventricular rate during recurrence.[3] |
| Dronedarone ERATO | Non-pivotal | Change in mean ventricular rate by 24-hour Holter recording on Day 14 (Dronedarone versus placebo) | Dronedarone 800 mg: mean -11 beats per minute (86.5 to 76.2); Placebo: mean +0.7 beats per minutes (90.6 to 90.2) | **Negative**:  Clinically significant secondary endpoints (change in maximal exercise duration) not affected. | **Positive**:  Improvement in ventricular rate.[4] |
| Dronedarone EURIDIS | Pivotal | Time between randomization and first atrial fibrillation/flutter recurrence (Dronedarone versus placebo) | Dronedarone 800 mg: 41 days; placebo: 96 days; log-rank test P valve 0.013 | **Equivocal**:  Delayed time to recurrence, but no clinically significant reduction in ventricular rate at first recurrence | **Positive**:  Delayed time to recurrence, and reduced ventricular rate during recurrence.[3] |
| Insulin Detemir 1205 | Non-pivotal | Mean change in hemoglobin A1c at 26 weeks (detemir versus NPH insulin) | A1c decreased 0.56% in determir group, 0.5% in NPH group (no significant difference between groups) | **Negative**:  “Nominally met” non-inferiority goal, but additional insulin aspart boluses used in Detemir group; Detemir and NPH “cannot be considered equally effective.” | **Positive**:  Detemir provided more predictable glycemic control, smoother plasma glucose profiles, and lower risk of hypoglycemia; detemir “may be able to improve glycemic control beyond that possible with NPH insulin.”[5] |
| Insulin Detemir 1335 | Non-pivotal | Mean change in hemoglobin A1c at 26 weeks (detemir versus NPH insulin) | No significant difference from baseline to 26 weeks in either group | **Negative**:  Neither Detemir nor NPH “effective in achieving good control,” glycemic goals not met. | **Positive**:  Less variability in fasting blood glucose (secondary outcome), ergo Detemir group achieved “stricter blood glucose control targets compared to NPH.”[6] |
| Insulin Detemir 1336 | Non-pivotal | Mean change in hemoglobin A1c at 26 weeks (detemir versus NPH insulin) | A1c decreased 0.26% in Detemir group, 0.36% in NPH group (ANOVA 0.16, 95% CI 0.003-0.312, non-inferiority margin met) | **Equivocal**:  Met non-inferiority, but patients on Detemir received additional insulin aspart mealtime boluses | **Positive**:  Met non-inferiority, and patients in Detemir group had less variability in fasting blood glucose and less weight gain.[7] |
| Insulin Detemir 1374 | Non-pivotal | Mean change in hemoglobin A1c at 18 weeks (determir plus aspart versus NPH plus regular insulin) | Mean A1c at endpoint lower in detemir plus aspart group by 0.22 (P=0.004) | **Negative**:  Effects of detemir (in comparison to NPH) cannot be separated from effects of aspart (in comparison to regular insulin) | **Positive**:  Detemir/aspart “better balance of control and tolerability.”[8] |
| Insulin Detemir 1385 | Non-pivotal | Mean change in hemoglobin A1c at 22 weeks (determir plus aspart versus NPH plus regular insulin) | Mean reduction of about 0.6% in both groups (criterion for superiority not met) | **Negative**:  Criterion for superiority not met. | **Positive**:  Similar changes in A1c, less weight gain and within-person fasting blood glucose variability in detemir group. |
| Insulin Detemir 1447 | Non-pivotal | Mean change in hemoglobin A1c at 16 weeks (two regimens of detemir versus NPH) | Mean reduction of 0.53%, 0.39%, 0.49% for detemir dinner, NPH, detemir bedtime groups, respectively (not statistically different) | **Negative**:  Did not clearly establish non-inferiority of Detemir relative to NPH (patients on detemir received more insulin) | **Positive:**  Detemir groups had lower and less variable glucose levels compared to NPH groups.[9] |
| Linagliptin 1218.20 | Non-pivotal | Change in hemoglobin A1c from baseline to week 104 (linagliptin versus glimepiride) | Mean reduction 0.65% for glimepiride, 0.43% for linaglpitin (non-inferiority margin met) | **Equivocal**:  “Clinical application limited” due to submaximal dose of glimepiride (trial dose 4 mg instead of 8 mg) | **Positive:**  Non-inferiority met, with additional benefits of less hypoglycemia, less weight loss, and fewer cardiovascular events.[10] |
| Linagliptin 1218.23 | Non-pivotal | Change in hemoglobin A1c at 12 weeks (versus placebo) and 26 weeks (versus voglibose) | Mean difference in reduction at 12 weeks 0.87% and 0.88% for linaglitin 5mg and 10 mg dose, respectively; at 26 weeks difference from voglibose was 0.32% and 0.39%, respectively | **Equivocal**:  Results have “limited usefulness” due to intervention group extending to 52 weeks without placebo control, and voglibose not marketed in U.S.A | **Positive**:  Linagliptin showed superior glucose lowering efficacy to both placebo and voglibose with a similar safety and tolerability profile.[11] |
| Pitavastatin 305 | Pivotal | Change in LDL cholesterol from baseline to 12 weeks (pitavastatin 4 mg versus atorvastatin 20 mg) | Reduction in LDL 40.8% in pitavastatin group, 43.3% in atorvastatin group; mean difference -0.23% with lower bound of 95% confidence interval -6.2 (below pre-specified target of -6) | **Negative:**  Lower bound of confidence interval below prespecified value, therefore pitavastatin “not non-inferior to atorvastatin.” | **Positive:**  Used post-hoc analysis to show that reduction in LDL similar in pitavastatin and atorvastatin groups, with possible better side effect profile of pitavastatin.[12] |
| Pramlintide 111 | Pivotal | Change in hemoglobin A1c from baseline to 52 weeks | Only significant difference from placebo was highest dose group (150 ug three times daily) at 52 weeks | **Equivocal**:  Reduction in hemoglobin A1c only shown at highest dose; lower doses not different than placebo | **Positive:**  Used secondary endpoint to show that reduction in hemoglobin A1c at 13 weeks (instead of 52 weeks) significant for both 75 ug and 150 ug dose groups.[13] |
| Ranolazine 072 | Non-pivotal | Peak exercise duration after administration of ranolazine or placebo | Significant improvement only in highest dose group (240 mg) versus placebo | **Equivocal**:  Significant findings only in highest dose group; no dose-response relationship | **Positive**:  Highlighted significant anti-anginal action in addition to exercise duration. |

**References**

1. Pool JL, Schmieder RE, Azizi M, Aldigier JC, Januszewicz A, Zidek W, Chiang Y, Satlin A: **Aliskiren, an orally effective renin inhibitor, provides antihypertensive efficacy alone and in combination with valsartan**. *American journal of hypertension* 2007, **20**(1):11-20.

2. Verdecchia P, Calvo C, Mockel V, Keeling L, Satlin A: **Safety and efficacy of the oral direct renin inhibitor aliskiren in elderly patients with hypertension**. *Blood pressure* 2007, **16**(6):381-391.

3. Singh BN, Connolly SJ, Crijns HJGM, Roy D, Kowey PR, Capucci A, Radzik D, Aliot EM, Hohnloser SH: **Dronedarone for Maintenance of Sinus Rhythm in Atrial Fibrillation or Flutter**. *New England Journal of Medicine* 2007, **357**(10):987-999.

4. Davy J-M, Herold M, Hoglund C, Timmermans A, Alings A, Radzik D, Van Kempen L: **Dronedarone for the control of ventricular rate in permanent atrial fibrillation: The Efficacy and safety of dRonedArone for The cOntrol of ventricular rate during atrial fibrillation (ERATO) study**. *American Heart Journal* 2008, **156**(3):527.e521-527.e529.

5. Vague P, Selam J-L, Skeie S, De Leeuw I, Elte JWF, Haahr H, Kristensen A, Draeger E: **Insulin Detemir Is Associated With More Predictable Glycemic Control and Reduced Risk of Hypoglycemia Than NPH Insulin in Patients With Type 1 Diabetes on a Basal-Bolus Regimen With Premeal Insulin Aspart**. *Diabetes Care* 2003, **26**(3):590-596.

6. Russell-Jones D, Simpson R, Hylleberg B, Draeger E, Bolinder J: **Effects of QD insulin detemir or neutral protamine Hagedorn on blood glucose control in patients with type I diabetes mellitus using a basal-bolus regimen**. *Clinical Therapeutics* 2004, **26**(5):724-736.

7. Haak T, Tiengo A, Draeger E, Suntum M, Waldhäusl W: **Lower within-subject variability of fasting blood glucose and reduced weight gain with insulin detemir compared to NPH insulin in patients with type 2 diabetes**. *Diabetes, Obesity and Metabolism* 2005, **7**(1):56-64.

8. Hermansen K, Fontaine P, Kukolja KK, Peterkova V, Leth G, Gall MA: **Insulin analogues (insulin detemir and insulin aspart) versus traditional human insulins (NPH insulin and regular human insulin) in basal-bolus therapy for patients with Type 1 diabetes**. *Diabetologia* 2004, **47**(4):622-629.

9. Pieber TR, Draeger E, Kristensen A, Grill V: **Comparison of three multiple injection regimens for Type 1 diabetes: morning plus dinner or bedtime administration of insulin detemir vs. morning plus bedtime NPH insulin**. *Diabetic Medicine* 2005, **22**(7):850-857.

10. Gallwitz B, Rosenstock J, Rauch T, Bhattacharya S, Patel S, von Eynatten M, Dugi KA, Woerle H-J: **2-year efficacy and safety of linagliptin compared with glimepiride in patients with type 2 diabetes inadequately controlled on metformin: a randomised, double-blind, non-inferiority trial**. *The Lancet*, **380**(9840):475-483.

11. Kawamori R, Inagaki N, Araki E, Watada H, Hayashi N, Horie Y, Sarashina A, Gong Y, von Eynatten M, Woerle HJ *et al*: **Linagliptin monotherapy provides superior glycaemic control versus placebo or voglibose with comparable safety in Japanese patients with type 2 diabetes: a randomized, placebo and active comparator-controlled, double-blind study**. *Diabetes, Obesity and Metabolism* 2012, **14**(4):348-357.

12. Gumprecht J, Gosho M, Budinski D, Hounslow N: **Comparative long-term efficacy and tolerability of pitavastatin 4 mg and atorvastatin 20–40 mg in patients with type 2 diabetes mellitus and combined (mixed) dyslipidaemia**. *Diabetes, Obesity and Metabolism* 2011, **13**(11):1047-1055.

13. Ratner RE, Want LL, Fineman MS, Velte MJ, Ruggles JA, Gottlieb A, Weyer C, Kolterman OG: **Adjunctive Therapy with the Amylin Analogue Pramlintide Leads to a Combined Improvement in Glycemic and Weight Control in Insulin-Treated Subjects with Type 2 Diabetes**. *Diabetes Technology & Therapeutics* 2002, **4**(1):51-61.
